# The Obituary of the Brazilian Amazon Entomologist: Nelson Ferreira Fé (★1941 †2023)

**DOI:** 10.1590/0037-8682-0260-2023

**Published:** 2023-07-24

**Authors:** Marcus Vinícius Guimarães Lacerda, Wuelton Monteiro, Adam Hendy, Marcus Vinitius de Farias Guerra, Maria das Graças Vale Barbosa Guerra

**Affiliations:** 1 Fundação de Medicina Tropical Doutor Heitor Vieira Dourado, Manaus, AM, Brasil. Fundação de Medicina Tropical Doutor Heitor Vieira Dourado Manaus AM Brasil; 2 Instituto Leônidas & Maria Deane, Fiocruz, Manaus, AM, Brasil. Instituto Leônidas & Maria Deane Fiocruz Manaus AM Brasil; 3 University of Texas Medical Branch, Galveston, TX, USA. University of Texas Medical Branch Galveston TX USA; 4 Universidade do Estado do Amazonas, Manaus, AM, Brasil. Universidade do Estado do Amazonas Manaus AM Brasil; 5 Fundação Hospitalar Alfredo da Matta, Manaus, AM, Brasil. Fundação Hospitalar Alfredo da Matta Manaus AM Brasil

Obituaries are usually published in scientific journals by close colleagues and distinguished researchers. After living a life of achievement, the death of a scientist is ultimately mourned in a written piece describing the *opera omnia* of the deceased. An obituary is the coronation of an interrupted life devoted to adding more to human knowledge. It may serve as the final overview of seminal discoveries, a description of how that fellow changed his field of expertise, and finally, as a manuscript to stimulate young researchers to discover their careers. Indeed, we all need an idol to inspire and keep us moving forward in a difficult and competitive niche.

Due to their unique capabilities, the death of some scientists may lead to gaps in certain areas of knowledge, especially if they leave no scientific descendency. The world is also filled with scientists who lack formal training, but as autodidacts, they change the world around them. Universities and graduate programs are relatively young in the history of humankind; however, they do not preclude those who lived before this time from being considered geniuses.

Some could consider Mr. Nelson Ferreira Fé a common man and a disciplined public servant. However, he was much more than that, and those who had the chance to spend their time close to him were fully aware of his genuine talent. The fortunate ones learned so much that calling him "Mr. Fé" sounds indelicate and imprecise. To us, he was a true professor.

**Figure:**
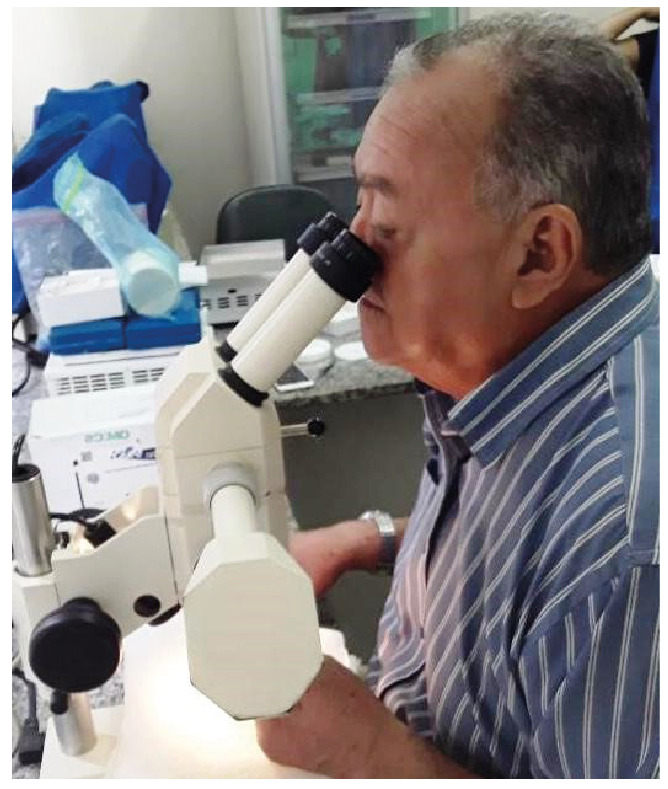

He was born in the state of Pará, in the Eastern Brazilian Amazon (Óbidos, June 23, 1941). Having worked as a microscopist in the Malaria Eradication Program, he came to know many experts in the field, such as Leônidas Deane, Maria Deane, Ítalo Sherlock, Heitor Dourado, Agostinho Marques, and many others. However, he had a particular interest in vector control and, over time, began to appreciate identifying medically important mosquitoes, biting flies, and other bugs.

Due to financial constraints, as a result of coming from a humble family, he never went to college, though he later went to Manaus as an employee for the *Superintendência de Campanhas de Saúde Pública* (SUCAM). Subsequently, he was invited by Heitor Dourado to join the recently founded *Instituto de Medicina Tropical de Manaus*, an institution run by the government of the state of Amazonas on the outskirts of the city, where outbreaks of malaria were being systematically reported. That institution became, over time, the reference center for infectious diseases in the Western Brazilian Amazon. He worked there until the last day of his life, leading the Entomology Laboratory for decades, where he had close contact with well-known entomologists worldwide, such as Don Roberts from the Uniformed Services University of Health Sciences (Bethesda, USA).

He was always invited to be part of the central core of scientists’ leading projects on malaria, leishmaniasis, Chagas disease, or dengue. While his lack of formal higher education prevented him from applying directly for project funding, his involvement and entomological expertise were key to the success of pivotal field studies at Rio Preto da Eva^1^, Iranduba^1^, Manacapuru^1^, Manaquiri^1^, Careiro^1^, Padauiri River^2^, Jaú River^3^, São Paulo de Olivença^4^, Barcelos^2^^,^^4^, Careiro Castanho^5^, São Gabriel da Cahoeira^5^, Manaus^6^^-^^16^, Cori and Tefé in Amazonas^17^, Serra do Navio^18^ in Amapá, Monte Dourado^18^ in Pará, Boa Vista in Roraima^19^, Peixoto de Azevedo^18^ in Mato Grosso^18^, Santa Elena de Uairém, State of Bolivar, Venezuela^18^ all of which were coordinated by renowned scientists.

Most professionals trained in Tropical Medicine in Manaus had the chance to spend some time in his laboratory, where he patiently magnified a mosquito and told us how to identify its gender and/or species. He shared and imprinted his patience, commitment, and a fine sense of humor on whoever wanted to delve deeper into the science of entomology.

Later on, as young scientists, we realized that he was also recognized and admired by many national and international research institutions and was frequently invited to participate in top-level projects from the University of São Paulo and *Fundação Oswaldo Cruz* (Fiocruz). However, given his expertise, he was seldom adequately remunerated and did not always share formal authorship in international publications, despite his committed work.

He was undoubtedly a much more competent technician. His skills in rapidly identifying vectors, based on in-depth knowledge of regional insect fauna and associated taxonomic keys, surpassed those of many PhD-level scientists who spent vast amounts of time and resources performing molecular analyses to confirm the identity of a given species or complex.

A lack of spoken and written English did not prevent him from collaborating with English-speaking researchers or studying English texts. Students and close admirers translated whenever needed so he could continue working in an up-to-date and precise manner.

In the field, he respected local populations, approaching families in their houses to inform them of his research and providing an overview of the tropical disease while paying equal attention to the parasite, its vector, and the ensuing clinical condition. He was proud to have had many episodes of leishmaniasis and countless more of malaria. He was often exposed to their bites while training others in the ‘human bait’ technique for collecting *Anopheles* mosquitoes. A couple of weeks later, he could be found shivering with confirmed malaria. This became an initiation ritual for those who, like him, had a passion for medical entomology.

He was the first to identify the phlebotomine sand flies *Lutzomyia (Psychodopygus) douradoi*^20^ and *Trichophoromyia uniniensis*^4^ and the scorpion *Opisthacanthus borboremai*^21^*,* and was honored in the description of a new species of scorpion named *Brotheochactas fei*^22^.

In one of the last commitments of his professional career, spanning the COVID pandemic and already suffering from diabetes and fighting cancer, he was critical to the success of a project funded by the National Institutes of Health (NIH) in partnership with the University of Texas Medical Branch (UTMB, Galveston, USA), which was devoted to identifying potential bridge vectors of mosquito-borne viruses in the rainforest bordering Manaus^8^^,^^9^^,^^15^^,^^16^. Despite his poor health, he continued to work with pride and was determined to complete what he had started.

In life, greatness is achieved through competency and social status in scientific societies. Despite the impossibility of formal membership, Mr. Fé chose the Brazilian Society of Tropical Medicine as his community. He rarely missed annual meetings and was undoubtedly a star in his field. He never received a degree. Many scientists with whom he worked never thought of awarding him the title of *Doctor Honoris Causa*. We all owe him so much that an obituary in his honor will never be enough to thank him for all his time and companionship.

Counting only a few manuscripts in which he is listed as a formal author because of the shameful belief that authors must have a degree, Mr. Fé still appears in 30 indexed papers^1^^-^^21^^,^^23^^-^^31^. 

The day before his departure (April 2^nd^, 2023), he was awarded a plaque from the director president of his beloved institution in the ward where he was hospitalized, as if awaiting some final recognition before leaving us, as if we had failed as colleagues and peers, denying him so many academic formalities when he was more talented than most of us.

Today, almost nobody thinks of death as an inevitability. We do not know if Mr. Fé knew that death was approaching, but even if he did, he would not stop thinking about the laboratory tasks. According to the French historian Phillipe Ariès, a striking feature of modern times is that death is not even considered by those who still have a lot of work to do^32^. On the eve of his departure, Mr. Fé was concerned about identifying triatomine bugs in the municipality of Apuí, in southern Amazonas, where a case of acute Chagas disease had been detected. In his last few days and hours, he did not hide from his colleagues and friends but left his door open, receiving as many people as possible to talk with as much strength as possible about the past and the future and, perhaps, to say farewell.

We all know that this obituary is not only about Nelson Ferreira Fé but also about the entomology studies he performed in the Amazon, as recognized by those born in this complex ecosystem. We have all lost a friend, but entomology has lost so much more.
